# Genomic epidemiology of highly pathogenic avian influenza A (H5N1) virus in wild birds in South Korea during 2021–2022: Changes in viral epidemic patterns

**DOI:** 10.1093/ve/veae014

**Published:** 2024-02-07

**Authors:** Ji-Yun Kim, Sol Jeong, Da-Won Kim, Dong-Wook Lee, Dong-Hun Lee, Daehun Kim, Jung-Hoon Kwon

**Affiliations:** College of Veterinary Medicine, Kyungpook National University, 80 Daehak-ro, Daegu 41566, Republic of Korea; Wildlife Disease Research Team, National Institute of Wildlife Disease Control and Prevention (NIWDC), Ministry of Environment, 1, Songam-gil, Gwangju 62407, Republic of Korea; College of Veterinary Medicine, Kyungpook National University, 80 Daehak-ro, Daegu 41566, Republic of Korea; College of Veterinary Medicine, Kyungpook National University, 80 Daehak-ro, Daegu 41566, Republic of Korea; College of Veterinary Medicine, Konkuk University, 120, Neungdong-ro, Seoul 05029, Republic of Korea; Wildlife Disease Research Team, National Institute of Wildlife Disease Control and Prevention (NIWDC), Ministry of Environment, 1, Songam-gil, Gwangju 62407, Republic of Korea; College of Veterinary Medicine, Kyungpook National University, 80 Daehak-ro, Daegu 41566, Republic of Korea

**Keywords:** avian influenza, phylogeny, genomic epidemiology, republic of korea, wild bird

## Abstract

Clade 2.3.4.4b highly pathogenic avian influenza A (HPAI) viruses have been detected in wild birds worldwide, causing recurrent outbreaks since 2016. During the winter of 2021–2022, we detected one H5N8 and forty-three H5N1 clade 2.3.4.4b HPAI viruses from wild birds in South Korea. Phylogenetic analysis revealed that HA gene of H5N1 viruses was divided into two genetically distinct groups (N1.G1 and N1.G2). Bayesian phylodynamic analysis demonstrated that wild birds play a vital role in viral transmission and long-term maintenance. We identified five genotypes (N1.G1.1, N1.G2, N1.G2.1, N1.G2.2, and N1.G2.2.1) having distinct gene segment constellations most probably produced by reassortments with low-pathogenic avian influenza viruses. Our results suggest that clade 2.3.4.4b persists in wild birds for a long time, causing continuous outbreaks, compared with previous clades of H5 HPAI viruses. Our study emphasizes the need for enhancing control measures in response to the changing viral epidemiology.

## Introduction

Wild waterfowls, Anseriformes, and Charadriiformes are natural hosts of low-pathogenic avian influenza viruses (LPAIV) (Webster et al. 1992). H5 and H7 subtype LPAIVs could evolve into highly pathogenic avian influenza (HPAI) viruses by the insertion of basic amino acids in the cleavage site of hemagglutinin (HA) protein during their adaptation in Gallinaceous poultry (Swayne, 2009). HPAI viruses (HPAIV) cause up to 100 per cent mortality in Galliformes, but various rates of mortality in Anseriformes (Swayne 2009). From 1959 to 2020, forty-three distinct HPAI epizootics have been detected and most outbreaks were geographically restricted and eradicated by national disease control policies (Youk et al. 2020; Lee, Criado, and Swayne 2021). Nevertheless, the A/goose/Guangdong/1/96 (Gs/GD) lineage has spread globally by wild birds and continuously caused outbreaks in birds and mammals since the first detection in China in 1996 (Global Consortium for H5N8 and Related Influenza Viruses 2016). The Gs/GD lineage H5Nx viruses have evolved into 10HA genetic clades (0–9) and their subclades (WHO/OIE/FAO H5N1 Evolution Working Group 2008).

Historically, wild birds have not been significantly involved in the epidemiology of HPAI, except for the Gs/GD lineage H5Nx viruses (Alexander 2007). Remarkably, long-distance transmission of some Gs/GD lineage H5Nx viruses by migratory birds had resulted in global HPAI outbreaks. A large outbreak of clade 2.2 H5N1 virus occurred in migratory waterfowl at Qinghai Lake, China, in 2005 and subsequently spread across Asia and into Europe (Tosh et al. 2014). Clade 2.3.2.1 and clade 2.3.4.4 viruses also resulted in global outbreaks by wild birds during 2010–2022 and 2014–2023, respectively. However, HPAIVs disappeared from wild birds within 1 year, with the remarkable exception of clade 2.3.4.4 viruses. Viruses of clade 2.3.4.4 have evolved into eight subgroups (a–h), as of 2022, according to phylogenetic distinction by the World Health Organization (World Health Organization 2022). Since 2016, clade 2.3.4.4b viruses have been repeatedly detected in wild birds and caused global outbreaks (Ndumu et al. 2018; Pohlmann et al. 2018; Yehia et al. 2018; Caliendo et al. 2022; Gu et al. 2022).

The first outbreak of clade 2.3.4.4b H5N8 HPAI was reported in domestic ducks in eastern China in 2010 (Wu et al. 2014). Novel reassortants containing five Eurasian LPAIV gene segments were identified from wild birds at Qinghai Lake, China, in May 2016, and at Uvs-Nuur Lake, Mongolia, in June 2016 (Li et al. 2017; Lee et al. 2017b). The viruses spread to Asia, Africa, Middle East, and Europe by wild birds and further reassorted with LPAI viruses, resulting in H5 HPAIVs with novel neuraminidase subtypes, including N1, N3, N4, N5, and N6 (Ndumu et al. 2018; Yehia et al. 2018; Poen et al. 2019; Grant et al. 2022; Gu et al. 2022). Since October 2020, a novel subtype of the clade 2.3.4.4b HPAIV, H5N1, has been detected in wild birds in Europe (Engelsma et al. 2022), which has since then disseminated worldwide, including North and South America (Caliendo et al. 2022; Jimenez-Bluhm et al. 2023).

In South Korea, two clade 2.3.4.4b H5N8 viruses were first detected in wild birds in January 2014 but disappeared from wild birds after a short outbreak (Lee et al. 2015). Since the global emergence and spread of the novel reassorted clade 2.3.4.4b HPAIVs in 2016, they have caused four HPAI outbreaks in South Korea, including 2016–2017 H5N8 (Woo et al. 2017), 2017–2018 H5N6 (Kwon et al. 2018), 2020–2021 H5N8 (Baek et al. 2021), and 2021–2022 H5N1/H5N8 (Sagong et al. 2022). During the 2021–2022 outbreak in Korea, HPAI outbreaks were caused by two distinct subtypes of clade 2.3.4.4b viruses, H5N1 and H5N8 (Sagong et al. 2022). However, the epidemiology of the 2021–2022 outbreak remains uncertain because of the limited availability of information. Further genome sequencing and investigation are required to elucidate the genetic characteristics and transmission patterns of these HPAIVs. Therefore, in this study, we isolated and sequenced forty-four HPAIVs from wild birds in South Korea during 2021–2022 winter. We conducted comparative phylogeographic analysis to determine the origin, evolution, and transmission pattern of clade 2.3.4.4b viruses. Moreover, we phylodynamically analyzed the contribution to the viral transmission of each host. Since HPAIVs have spread globally via wild birds, this study incorporates data analysis from a broader international context.

## Materials and methods

### Sample collection and virus isolation

Oropharyngeal and cloacal swabs of captured birds, carcasses of birds, and fecal samples were collected during national wild bird surveillance for avian influenza (AI) viruses by the National Institute of Wildlife Disease Control and Prevention (NIWDC), South Korea (Supplementary Table S1). Samples were screened for the matrix gene of AI virus by real-time reverse transcription-PCR (rRT-PCR) as described previously (Spackman et al. 2003). Influenza A virus-positive samples were inoculated into 10-day-old specific-pathogen-free embryonated chicken eggs and incubated for 72 h at 37°C. Harvested allantoic fluids were tested for hemagglutination activity using 10 per cent chicken red blood cells. RNA was extracted from the hemagglutinating-activity-positive allantoic fluid using the RNeasy Mini Kit (Qiagen, Hilden, Germany) according to the manufacturer’s instruction. The host of each sample positive for AIV was identified using a DNA barcoding technique based on cytochrome oxidase I gene, as described previously (Spackman 2014).

### Genome sequencing

The whole genome of HPAIVs was subjected to next-generation sequencing. The eight gene segments were amplified by PCR using Optil-F1, Optil-F2, and Optil-R1 primers as described previously (Lee 2020). The nucleotide sequences of the PCR products were analyzed using Nextera™ DNA Flex Library Prep Kit and the MiSeq system (Illumina, San Diego, CA, USA). The assembly of genome sequences was performed using the CLC Genomics Workbench software (Qiagen). The nucleotide sequences of each virus were deposited into the Global Initiative for Sharing All Influenza Data (GISAID) database (https://gisaid.org/) (Supplementary Table S1).

### Sequence dataset

The reference full-length genome sequences used in the phylogenetic analysis were obtained from the GISAID database. We downloaded 2,724 AIV subtype H5 sequences of HA gene collected during 2010–2022. Multiple sequence alignment was accomplished using the MAFFT version 1.5.0. Maximum likelihood (ML) tree was reconstructed using the RAxML version 8.2.11 with the general time reversible (GTR) substitution model and 500 bootstrap replicates (Stamatakis 2014). Descendants sharing a common node with A/duck/Jiangsu/K1203/2010 were extracted as clade 2.3.4.4b. We divided the sequences as eighteen groups according to the geographical location and host species, i.e. six geographical groups (Africa, Asia, Central Asia, Europe, Middle East, and North America) and three host species groups (domestic Anseriformes, domestic Galliformes, and wild birds). For effective computation, the number of sequences was reduced to 680 based on the sequence identity using the program CD-HIT (Li and Godzik 2006). To prevent unwanted sampling bias, data reduction was conducted separately for each group.

### Phylogenetic analysis

Time-scaled maximum clade credibility (MCC) tree of the HA gene was constructed using BEAST version 1.10.4 (Suchard et al. 2018) with 724 HA sequences, including 44 viruses from this study. The GTR nucleotide substitution and uncorrelated lognormal relaxed molecular clock models were selected as flexible approaches to estimate the substitution rate. A non-parametric tree model, GMRF Bayesian skyride coalescent tree prior, was used to estimate changes in the viral population size. For the phylodynamic analysis, the taxa were coded by their geographical location (Africa, Asia, Central Asia, Europe, Middle East, and North America), host species (domestic Anseriformes, domestic Galliformes, and wild birds), and subtype (H5N1, H5N2, H5N3, H5N4, H5N5, H5N6, H5N7, and H5N8). The ancestral location, host, and subtype were reconstructed for each ancestral node, and the asymmetric viral exchange was estimated using a non-reversible continuous-time Markov chain model. We also used the Bayesian stochastic search variable selection model to determine the statistical significance of each transition by the Bayes factor (BF) test. The Markov chain Monte Carlo was run in parallel for three chains, with 150 million steps. The parameters and trees were sampled every 150,000 steps.

The MCC tree was visualized by FigTree 1.4.2 (http://tree.bio.ed.ac.uk/software/figtree/) and SpreaD3 version 0.9.7.1 (Bielejec et al. 2016). Lines with posterior probabilities of ≥0.5 are indicated in the visualizations. Moreover, we investigated the contribution of each host species to the transmission dynamics of viruses. A transition of viruses between host types was estimated using a discrete ancestral state reconstruction method and asymmetric host transitions. The BF and posterior probability were calculated using SpreaD3 v0.9.7.1 and were considered significant when BF was >3 and posterior probability was >0.5. In addition, the number of transitions between host types (Markov jump) and the times between host type changes (Markov reward) were calculated (Minin and Suchard, 2008).

### Whole-genome phylogenetic and reassortment analysis

For genotyping, we downloaded the full genome sequences of AIVs isolated during 2016–2022 if all the eight gene segments were available in the GISAID database. Redundant sequences were removed using CD-HIT. ML trees were constructed using 272 sequences for polymerase basic gene 2 (PB2), polymerase basic gene 1 (PB1), polymerase acidic gene (PA), nucleoprotein gene (NP), and matrix gene (M); 252 sequences for non-structural gene (NS); 224 sequences for HA subtype 5; and 198 sequences for neuraminidase gene (NA) subtype 1 using RAxML version 8.2.11, as described previously (Stamatakis 2014). ML trees were visualized using FigTree 1.4.2.

Tanglegrams were generated using the Baltic model (https://github.com/evogytis/baltic) to visualize the dynamics of reassortment events. Rooted phylogenetic tree data were fed into the Baltic model in the Newick format.

## Results

### Virus isolation

**Figure 1.**
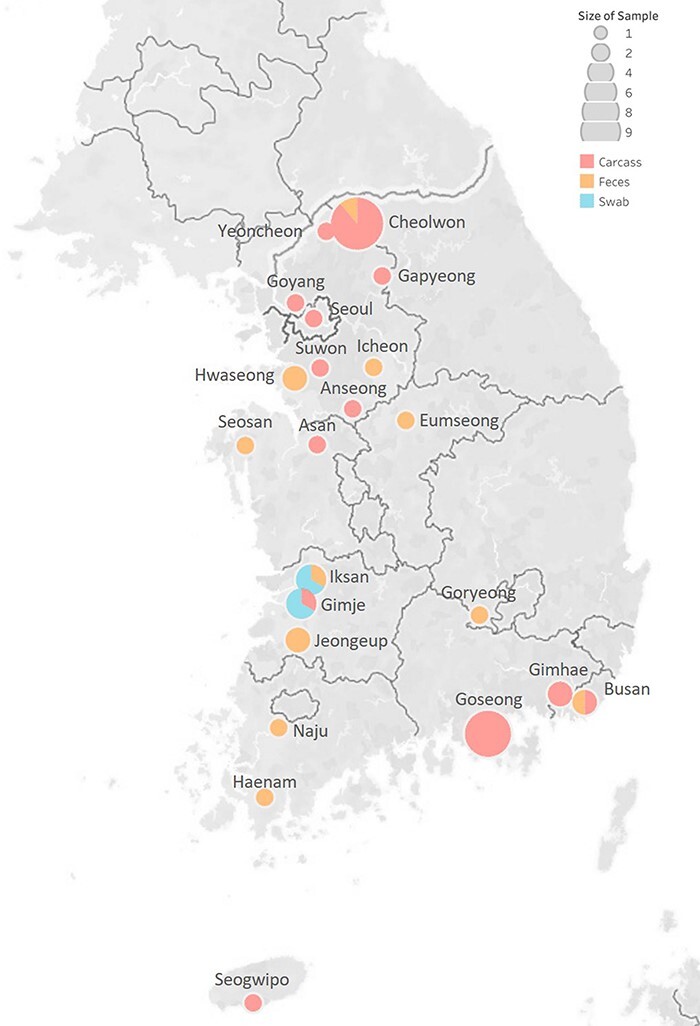
The location of the virus isolated and the type of sample visualized on the map.

During November 2021–March 2022, forty-three subtype H5N1 HPAIVs and 1 subtype H5N8 HPAIV were isolated from wild birds (thirteen fecal droppings, twenty-seven carcasses, and four swab samples of captured birds) in South Korea (Fig. 1, Supplementary Table S1). These isolates were identified as HPAIVs harboring multiple basic amino acids (PLREKRRKR/G [*n* = 4], PLRERRRKR/G [*n* = 40]) within the cleavage site of the HA gene, indicating a high-pathogenicity phenotype in chickens.

### Phylogenetic analysis of HA gene of South Korean H5Nx isolates during 2021–2022 winter

**Figure 2.**
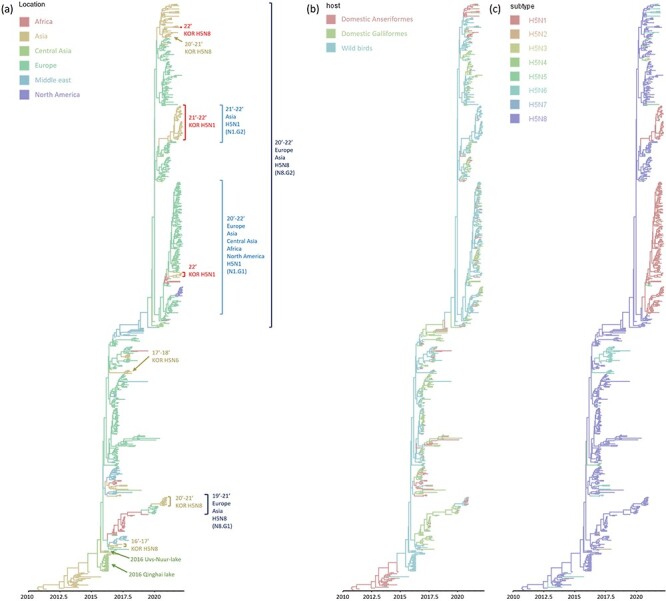
Time-scaled MCC tree-based Bayesian analysis of HA gene segment of 2.3.4.4b HPAI viruses. Each tree branch was colored according to three traits: (A) geographical locations, (B) host, and (C) subtype.

Phylogenetic analysis revealed that all forty-four viruses isolated from wild birds in South Korea during 2021–2022, including forty-three H5N1 and one H5N8, belonged to clade 2.3.4.4b virus and the lineage originated from wild birds in Uvs-Nuur Lake and Qinghai Lake (Fig. 2, Supplementary Figure S1). The H5N8 viruses that caused outbreaks in Korea during 2020–2021 were divided into two subgroups having a distinct HA gene, G1 and G2 (hereafter, N8.G1 and N8.G2, respectively) (Fig. 2a) (Baek et al. 2021). All viruses isolated in this study clustered with the N8.G2 subgroup and are genetically distinct from HPAIVs that caused outbreaks in Korea during 2016–2018. The forty-three 2021–2022 H5N1 viruses were divided into two genetically distinct clusters. Four isolates clustered with viruses isolated primarily in Europe during 2021–2022 (N1.G1). The thirty-nine viruses clustered with viruses isolated primarily from East Asia, including China and South Korea (N1.G2). The A/Whooper swan/Korea/21WC116/2022 (H5N8) virus clustered with H5N8 viruses isolated from China, Mongolia, South Korea, and Vietnam during 2020–2021 winter. The genetically closest virus was A/chicken/Korea/H008/2021(H5N8). Based on these results, we assume that H5N8 viruses have continuously circulated in Asia undetected for more than a year.

We used a Bayesian skyride plot to evaluate the population growth over time. Overall, the population size, which indicates the relative genetic diversity of clade 2.3.4.4b, exhibited an increasing trend from 2012 to 2022. A sharp increase occurred during the 2013–2014 winter followed by genetic stabilization during 2014–2015. The next rapid increase occurred between early 2016 and late 2017 corresponding with the first detection of novel reassortants. The third increase occurred from late 2020 after a moderate decrease in genetic diversity during 2018–2019. The final peak occurred in late 2021 (Supplementary Figure S2).

### Global phylogeographic analysis revealed the global spread of clade 2.3.4.4b viruses

**Figure 3.**
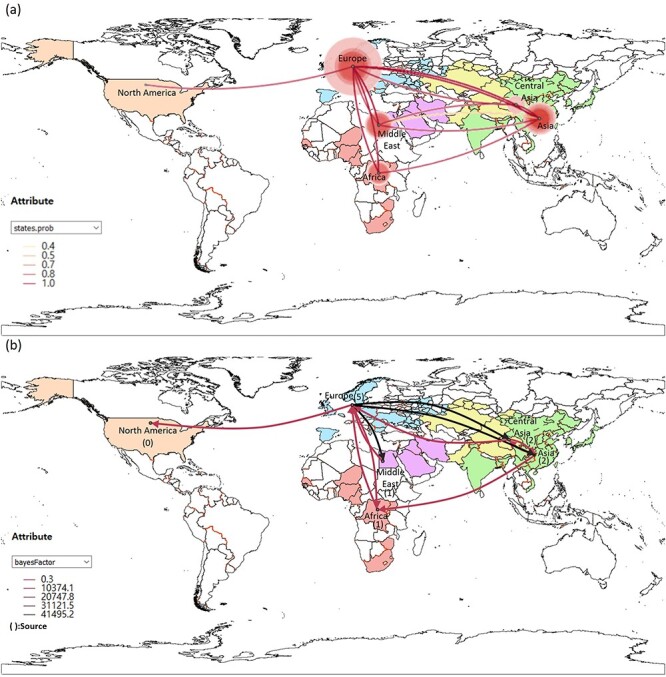
Visualization of the phylogeographic transmission pathway on the global map. The visualization of the transmission is derived from the data presented in Table 1. (A) Transmission of the H5 hemagglutinin gene of the highly pathogenic avian influenza clade 2.3.4.4b viruses. Size of Circles represents the count of emergence. The lines were colored according to the probability of each transition. (B) Transmission lines with a posterior probability of ≥0.5 are shown with darker color representing a higher Bayes factor. The arrows represent the direction of transmission. The world map was colored corresponding to the geographical groups used in this study.

The phylogeographic analysis revealed that clade 2.3.4.4b HPAI started spreading globally after the first detection of the novel reassorted clade 2.3.4.4b HPAI in Central Asia in 2016 (Fig. 3, Video S1). After its emergence in Central Asia, clade 2.3.4.4b virus spread worldwide. The viral transitions from Europe to all other regions and the transition from Africa to Europe were supported by high BF and posterior probability values (BF: >100, posterior probability: >0.9) (Table 1). The most frequent transition was detected from Europe to Central Asia, with the highest transition rate (2.392) and Markov jump count (14.626) (Table 1). Other transmission routes, supported by a high BF, were detected between geographically close regions sharing wild bird migratory routes, except for the route from East Asia to Africa (Table 1). The transmission from North America to other regions was not detected in this study.

**Table 1.** Viral transition rates and numbers between geographical locations and their statistical support values for the H5 hemagglutinin gene of the highly pathogenic avian influenza clade 2.3.4.4b viruses isolated worldwide during 2010–2022.

Transition from	Transition to	Mean actual transition rate^a^ (95% BCI)	Mean number of Markov jumps (95% BCI)	Bayes factor	Posterior probability
Africa	Europe	1.221 (0.058–2.806)	2.322 (1–4)	>200	0.997
Asia^b^	Africa	0.373 (0–1.322)	0.854 (0–2)	7.217	0.628
	Central Asia	0.514 (0–1.489)	1.357 (0–3)	13.138	0.755
Central Asia	Asia	1.139 (0–2.785)	2.322 (1–6)	44.611	0.913
	Europe	1.149 (0–3.105)	2.339 (0–8)	17.864	0.807
Europe	Africa	0.738 (0.135–1.692)	4.272 (3–6)	>200	0.999
	Asia	1.569 (0.391–3.040)	9.641 (6–14)	>200	1.000
	Central Asia	2.392 (0.706–4.392)	14.626 (9–20)	>200	1
	Middle East	1.273 (0.233–2.427)	7.663 (5–10)	>200	1
	North America	0.293 (0–0.744)	1.055 (1–2)	123.815	0.967
Middle East	Central Asia	0.363 (0–1.448)	1.129 (1–2)	4.499	0.513
	Europe	0.832 (0–2.077)	1.201 (0–3)	41.334	0.906

a Actual transition rates were calculated as rate × indicator.

b Asia regions excluding West and Central Asia.

As no isolates were detected between August and December 2019, we inferred the ancestral location of 2020–2022 viruses by phylogeographic analysis, which indicated that the most probable ancestral location of the 2020–2022 H5N8 (N8.G2) and H5N1 viruses is the Middle East (posterior probability: 0.939). The ancestor location of the other 2020–2021 H5N8 virus group N8.G1 was estimated as Africa (posterior probability: 0.972) (Fig. 2a). Both the N8.G1 and N8.G2 groups transmitted to Europe and subsequently spread to other regions.

### Host phylodynamic analysis revealed long-term circulation in wild birds

**Figure 4.**
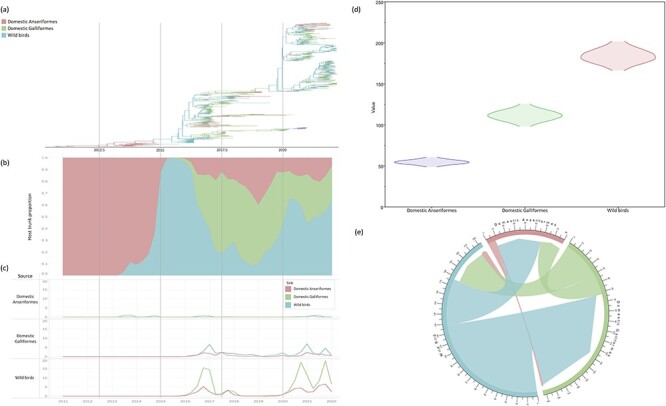
Host transition and MCC tree for H5 hemagglutinin gene of the highly pathogenic avian influenza clade 2.3.4.4b viruses. Statistical data related to the transition are displayed in Table 2. (A) MCC tree colored by host type. (B) The proportion of the trunk belonging to each host type over time. (C) The chart in line format shows the number of transition events between each host type. (D) The violin plot of each host type (X-axis) depicts the density distribution of the total time spent in years (Y-axis). (E) Illustration showing the transition between each host type. Line thickness corresponds to Markov jump counts.

We investigated the role of wild birds in the viral spread by phylodynamic analysis between host species. The transition rate, number of transitions (Markov jump count), and the statistical supporting value of the transition (posterior probability and BF) were estimated (Table 2, Fig. 4). We observed the highest mean actual migration rate and number of Markov jumps for the transmission from wild birds to domestic Galliformes (migration rate: 2.523, Markov jumps: 128.448), indicating frequent viral transmission from wild birds to poultry farms worldwide. The transmissions from wild birds to poultry were constantly identified during the epizootic period, i.e. late 2016, late 2020, and late 2021 (Fig. 4c). The transmission from domestic Galliformes to wild birds was also detected during the same periods, but the number of transmissions was relatively lower than that from wild birds to domestic Galliformes (Fig. 4c). A low number of transmissions was also detected from domestic Galliformes to domestic Anseriformes, but transmissions from domestic Anseriformes to other species were rarely detected (Fig. 4c).

**Table 2.** Viral transition rates and numbers between host species and their statistical support values for the H5 hemagglutinin gene of the highly pathogenic avian influenza clade 2.3.4.4b viruses isolated worldwide during 2010–2022.

Transition from	Transition to	Mean actual migration rate^a^ (95% BCI)	Mean number of Markov jumps (95% BCI)	Bayes factor	Posterior probability
Domestic Anseriformes	Domestic Galliformes	0.041 (0–0.213)	2.282 (1–4)	0.646	0.345
	Wild birds	0.278 (0.031–0.602)	6.213 (4–10)	>200	0.999
Domestic Galliformes	Domestic Anseriformes	0.561 (0.094–1.126)	22.860 (14–31)	>200	1.0
	Wild birds	1.085 (0.211–2.124)	39.674 (25–56.5)	>200	1.0
Wild birds	Domestic Anseriformes	0.906 (0.224–1.790)	48.814 (40–58)	>200	1.0
	Domestic Galliformes	2.523 (0.509–4.651)	128.448 (109–148)	>200	1.0

a Actual transition rates were calculated as rate × indicator.

The time spent (Markov reward) by the virus in each host species was estimated by phylogeographic analysis. The estimated mean Markov reward time was highest in wild birds (183.903; 95 per cent Bayesian credibility interval (BCI): 166.287–201.758), followed by domestic Galliformes (111.561; 95 per cent BCI 98.214–125.4443), and domestic Anseriformes (54.710; 95 per cent BCI: 48.812–60.585), indicating the long-term maintenance of viruses in wild birds (Fig. 4d). These data suggest that wild birds played a vital role in the spread and maintenance of clade 2.3.4.4b viruses.

### Reassortment with LPAI viruses

**Figure 5.**
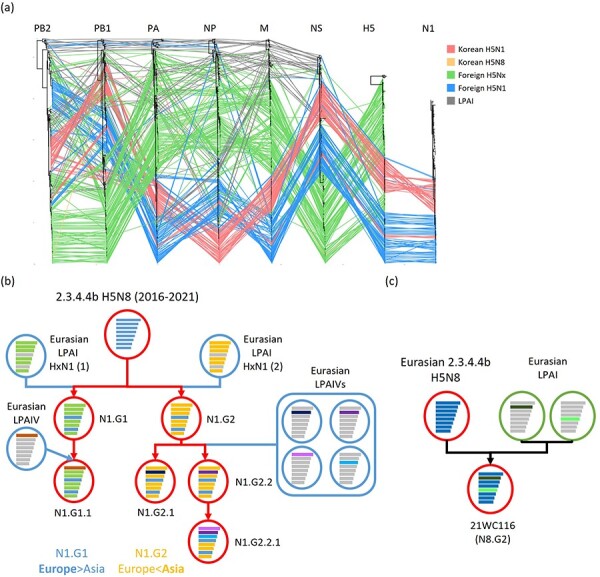
Reassortment of clade 2.3.4.4b viruses isolated in this study. (A) Tanglegram for each AIV gene segment. Connecting lines are colored according to the five groups. (B) Reassortment dynamics of H5N1 viruses. (C) Reassortment dynamics of a H5N8 virus. Each gene segment is represented by a bar of different length, from top to bottom: polymerase basic protein 2 (PB2), polymerase basic protein 1 (PB1), polymerase acidic protein (PA), hemagglutinin (HA), nucleoprotein (NP), neuraminidase (NA), matrix protein (M), and non-structural protein (NS).

The phylogenetic analysis of all gene segments revealed that multiple distinct genotypes having different genome constellations emerged by reassortment with LPAIVs (Fig. 5, Supplementary Figure S3). In total, forty-three H5N1 HPAIVs isolated in South Korea during the 2021–2022 winter were classified into five distinct genotypes, viz., N1.G1.1, N1.G2, N1.G2.1, N1.G2.2, and N1.G2.2.1 (Fig. 5b). The N1 gene was divided into two distinct clusters in the phylogenetic tree, and each cluster perfectly matching the HA gene subgroups N1.G1 and N1.G2 genotype, respectively (Fig. 5a). The N1.G1 genotype was mainly detected in Europe, and the N1.G2 genotype was mainly detected in Asia. The phylogenetic analysis demonstrated that these genotypes evolved through additional genetic reassortment. The N1.G1.1 genotype shared all the gene segments with N1.G1, except PB2, which clustered with other LPAIVs. The N1.G2.1 and N1.G2.2 genotypes contained a different PB1 gene from that in the N1.G2 genotype. The N1.G2.2.1 genotype had different PB2 and PA genes originating from LPAIVs compared with those in the N1.G2 genotype. The H5N8 HPAIV(21WC116) had different PB1 and NP genes compared with those in 2020–2021 H5N8 viruses detected in South Korea (Fig. 5c).

We estimated the tMRCA of each genotype to determine the emergence time of reassortants (Supplementary Table S2). Molecular dating analyses revealed the tMRCA of each genotype as follows: N1.G1, November 2019–September 2020; N1.G1.1, July 2021 (95 per cent HPD: February 2021–November 2021); N1.G2, August 2020–January 2021; N1.G2.1, December 2020 (95 per cent HPD: June 2020–June 2021); and N1.G2.2, June 2019 (95 per cent HPD: September 2021–January 2022).

## Discussion

During the four previous HPAI outbreaks in South Korea, clade 2.5 in 2003–2004, clade 2.2 in 2006–2007, clade 2.3.2 in 2008, and clade 2.3.2.1 in 2010–2011 (I-p et al. 2016), the HPAI outbreaks sustained for only 1–4 months. However, clade 2.3.4.4 viruses caused longer outbreaks than previous outbreaks caused by other HPAI clades. The clade 2.3.4.4c HPAI outbreak persisted for 28 months, from January 2014 to May 2016, in South Korea and continuously circulated in wild birds for more than 1 year (Kwon et al. 2016; Lee et al. 2017a). Clade 2.3.4.4c viruses spread westward to Europe and northeastward to North America during the 2014–2015 winter and were detected until August 2016 in North America (Lee et al. 2017c). After the spread of clade 2.3.4.4b viruses to wild birds, these viruses were detected for 22 months in wild birds, from May 2016 to March 2018 (Lee et al. 2017a). Despite the absence of wild bird cases until August 2020, related strains reemerged in September 2020, and the virus was maintained in wild birds for more than 28 months (as of February 2023). Our data reveal that clade 2.3.4.4b infection sustained in wild birds and caused outbreaks continuously for a longer period than previous outbreaks caused by other clades. Specifically, the H5N8 subtype virus identified in 2021–2022 in South Korea exhibited a close phylogenetic relationship with 2020–2021 viruses without detection in other countries, indicating a possible circulation of the virus in terrestrial wild birds or domestic birds for more than 1 year. Recently, an outbreak of clade 2.3.4.4b HPAI (H5N1) was reported in North America (Caliendo et al. 2022). Furthermore, unlike the previous intercontinental spread of 2.3.4.4c viruses through the Bering Strait region within the Pacific Flyway, clade 2.3.4.4b viruses spread from Europe to North America through the Atlantic Flyway (Caliendo et al. 2022). Reassorted Eurasian–North American H5N1 viruses were first reported in November 2022 in South America (Jimenez-Bluhm et al. 2023). These data indicate that the range and route of viral transmission through wild birds, particularly those of clade 2.3.4.4b viruses, have been changing.

Host transition analysis suggested that wild birds have played an important role in the transmission of clade 2.3.4.4b viruses and introducing them into South Korea. Our phylodynamic analysis inferred the frequent spread from wild birds to poultry and the long-term maintenance of the virus in wild birds. Between August 2019 and December 2019, when the viruses were not detected in wild birds, our data suggest that clade 2.3.4.4b viruses were sustained in poultry in Africa and the Middle East and then transmitted to wild birds. Nonetheless, we could not exclude the possibility that the viruses persisted in wild birds, because of the relatively low sampling frequency for wild birds in Africa and the Middle East. Before the emergence of clade 2.3.4.4b, HPAIV was circulated mainly in China where domestic Anseriformes played a key role in viral transmission, and unidirectionally introduced from China into South Korea through wild birds. As clade 2.3.4.4b adapted to wild birds, the source of the virus entering Korea is not limited to China (Baek et al. 2021; Kang et al. 2023).

We assume that the changed pattern of outbreaks, long-term circulation, and widespread transmission by wild birds could be caused by the adaptation of viruses in wild birds. According to previous studies, H5N1, H5N6, and H5N8 clade 2.3.4.4b viruses exhibited higher adaptation in domestic ducks than in chickens, suggesting efficient replication and spread in waterfowl species without clinical signs (Kim et al. 2014; Seekings et al. 2021; James et al. 2023). As the 2.3.4.4 H5Nx viruses adapted and maintained in wild birds, there could be more opportunities for spillover to domestic poultry. In this study, the viral spread between wild birds and domestic Galliformes was more frequent than that between wild birds and domestic Anseriformes. This is probably because most countries have a larger chicken industry than duck or goose industry. Previous studies have indicated that domestic Anseriformes played a major role in the local spread of the virus in countries having large duck industries, including China, France, Hungary, and South Korea (Kwon et al. 2020; Napp et al. 2018; Bi et al. 2016).

Following the spillover of the virus to wild birds, a wide variety of reassorted viruses containing the genes of LPAIVs in wild birds have been reported, including this study. We detected five different H5N1 genotypes in Korea that were produced by multiple reassortments with LPAIVs. The frequent reassortment with wild bird LPAIVs also supports our result that clade 2.3.4.4b viruses have continuously circulated in wild birds (Pohlmann et al. 2018; Poen et al. 2019; Baek et al. 2021). Because viral reassortment can cause rapid changes in the biological characteristics of viruses (Su et al. 2015), their rapid evolution by reassortment could cause infections in mammals. Recently, clade 2.3.4.4b H5N1 viruses were isolated from free-living mesocarnivores in America and European grey seals in Europe which contain a mutation that can affect receptor binding affinity to mammalian host (Alkie et al. 2023; Mirolo et al. 2023). The virus, which has a combination of mammalian adaptation mutations, can increase the risk of human infection. Human infection cases were already reported in Russia, China, and Ecuador in 2020, 2021, and 2023 (Pyankova et al. 2021; Gu et al. 2022; WHO 2023). However, the case of human infection of clade 2.3.4.4b is much fewer than previous Gs/GD-lineage viruses and WHO also still assess the risk of human infection as low (Assessment RR 2022). Despite this, the growing number of infection cases in non-human mammals, including sea lion infection cases in South America and domestic cat infection cases in North America, South Korea, and Poland (Domańska-Blicharz et al. 2023; Gamarra-Toledo et al. 2023; World Organisation for Animal Health 2023), highlights the need for continuous monitoring of novel mutants and reassortants.

Phylodynamic analysis can be influenced by sampling bias due to different sampling and sequencing frequencies in each country. Moreover, subsampling to reduce the number of sequences is unavoidable for an effective computation. Each country operates its own distinct influenza virus surveillance system. Genomic surveillance of wild birds is insufficient in Africa, Central Asia, and the Middle East. These factors act as inherent biases in the reconstruction of viral transition. Subsampling was conducted for each region and host type group separately to prevent additional sampling bias during the process. Despite our attempt to minimize bias, we cannot exclude the possibility that unsampled populations in certain geographic locations played a vital role in viral maintenance and transmission. For instance, wild birds traversing the Middle East and Africa may play a more significant role than that estimated in this study.

Overall, clade 2.3.4.4b viruses have caused continuous epidemics globally since 2016. Our phylodynamic analyses suggest that the viral introduction route to South Korea has changed from the past and wild birds primarily contribute to viral transmission and maintenance. Because the epidemiology of clade 2.3.4.4b HPAI has changed compared with that of previous HPAIs, different control strategies should be implemented to protect the poultry industry. It is essential to prepare countermeasures against enzootic situations in wild birds. Vaccination could serve as a method to prevent enzootic diseases, and some countries have begun preparing for emergency HPAI vaccination (EU 2023). However, enhancing biosecurity measures to block contact with wild birds or their excreta in poultry farms, especially those located near wild bird habitats, should be the primary approach.

## Supplementary Material

veae014_Supp

## Data Availability

All the sequences used in the study were deposited to the GISAID (https://gisaid.org/), and the accession numbers (EPI numbers) were annotated in Supplementary Table S1.
