# Supplementary material for: Genomic epidemiology of highly pathogenic avian influenza A (H5N1) virus in wild birds in South Korea during 2021–2022: Changes in viral epidemic patterns
Source: Virus Evol. 2024 Feb 7;10(1):veae014. doi: 10.1093/ve/veae014 (PMC10919474; doi:10.1093/ve/veae014)
Supplement: veae014_Supp [file veae014_supp.zip › suppl_data/Supplementary_figures_and_tables_revision_clean.docx]

Supplementary data

**Title: Genomic Epidemiology of Highly Pathogenic Avian Influenza A (H5N1) Virus in Wild Birds in South Korea during 2021–2022: Changes in Viral Epidemic Patterns**

Supplementary Table 1. List of highly pathogenic avian influenza viruses isolated in this study and their metadata.

| Virus name | Collection date | Sample type | Collection location | EPI accession number |
| --- | --- | --- | --- | --- |
| A/Mandarin duck/Korea/21WF119-1/2021 | 2021-11-15 | Feces | Bogha River, Icheon | EPI_ISL_18373217 |
| A/Mandarin duck/Korea/21WF91-6/2021 | 2021-11-16 | Feces | Banwolje Lake, Naju | EPI_ISL_18373218 |
| A/Wild duck/Korea/21WF124-4/2021 | 2021-11-22 | Feces | Geumjeong Reservoir, Eumseong | EPI_ISL_18373219 |
| A/Baikal teal/Korea/21WF192-9/2021 | 2021-12-01 | Feces | Geumho Lake, Haenam | EPI_ISL_18373220 |
| A/Wild duck/Korea/21WF241-1/2021 | 2021-12-05 | Feces | Ganwol Lake, Seosan | EPI_ISL_18373221 |
| A/Wild duck/Korea/21WF213-1/2021 | 2021-12-06 | Feces | Dongjin River, Jeongeup | EPI_ISL_18373222 |
| A/Wild duck/Korea/21WF225-24/2021 | 2021-12-07 | Feces | Nakdong River, Goryeong | EPI_ISL_18373223 |
| A/Wild bird feces/Korea/21WF312-4/2021 | 2021-12-22 | Feces | Nackdong River, Busan | EPI_ISL_18373264 |
| A/Eurasian wigeon/Korea/21WS24-27/2022 | 2022-01-17 | Swab | Mangyoeng River, Iksan | EPI_ISL_18373225 |
| A/Eurasian wigeon/Korea/21WS25-23/2022 | 2022-01-18 | Swab | Mangyoeng River, Iksan | EPI_ISL_18373226 |
| A/Spot-billed duck/Korea/21WS26-3/2022 | 2022-01-18 | Swab | Mangyoeng River, Gimje | EPI_ISL_18373227 |
| A/Eurasian wigeon/Korea/21WF549-3/2022 | 2022-01-19 | Feces | Mangyoeng River, Iksan | EPI_ISL_18373228 |
| A/Wild duck/Korea/21WF537-37/2022 | 2022-01-21 | Feces | Cheorwon Plain, Cheorwon | EPI_ISL_18373229 |
| A/Whooper swan/Korea/21WC116/2022 | 2022-01-23 | Carcass | Nackdong River, Busan | EPI_ISL_18373230 |
| A/Ruddy shelduck/Korea/21WC127/2022 | 2022-01-25 | Carcass | Angseongcheon Stream, Anseong | EPI_ISL_18373231 |
| A/Spot-billed duck/Korea/21WC138/2022 | 2022-01-25 | Carcass | Haebancheon Stream, Gimhae | EPI_ISL_18373232 |
| A/Wild duck/Korea/21WF573-1/2022 | 2022-01-26 | Feces | Geumgokcheon Stream, Hwaseong | EPI_ISL_18373233 |
| A/Spot-billed duck/Korea/21WC143/2022 | 2022-01-27 | Carcass | Haebancheon Stream, Gimhae | EPI_ISL_18373234 |
| A/White-fronted goose/Korea/21WC146/2022 | 2022-01-28 | Carcass | Cheorwon Plain, Cheorwon | EPI_ISL_18373235 |
| A/Wild duck/Korea/21WF563-5/2022 | 2022-01-25 | Feces | Hwanggujicheon Stream, Hwaseong | EPI_ISL_18373236 |
| A/White-fronted goose/Korea/21WC158/2022 | 2022-02-03 | Carcass | Cheorwon Plain, Cheorwon | EPI_ISL_18373237 |
| A/Mallard/Korea/21WC183/2022 | 2022-02-07 | Carcass | Ong Reservoir, Gimje | EPI_ISL_18373238 |
| A/Gadwall/Korea/21WC196/2022 | 2022-02-04 | Carcass | Ojo-ri, Seogwipo | EPI_ISL_18373239 |
| A/Bean goose/Korea/21WC198/2022 | 2022-02-10 | Carcass | Chukmanjae, Suwon | EPI_ISL_18373240 |
| A/White-fronted goose/Korea/21WC203/2022 | 2022-02-08 | Carcass | Cheorwon Plain, Cheorwon | EPI_ISL_18373241 |
| A/White-fronted goose/Korea/21WC209/2022 | 2022-02-10 | Carcass | Cheorwon Plain, Cheorwon | EPI_ISL_18373242 |
| A/White-fronted goose/Korea/21WC223/2022 | 2022-02-15 | Carcass | Cheorwon Plain, Cheorwon | EPI_ISL_18373243 |
| A/Mallard/Korea/21WS41-5/2022 | 2022-02-16 | Swab | Wonpyeongcheon Stream, Gimje | EPI_ISL_18373244 |
| A/White-fronted goose/Korea/21WC244/2022 | 2022-02-21 | Carcass | Cheorwon Plain, Cheorwon | EPI_ISL_18373245 |
| A/Great egret/Korea/21WC249/2022 | 2022-02-15 | Carcass | Gokgyocheon Stream, Asan | EPI_ISL_18373246 |
| A/White-naped crane/Korea/21WC257/2022 | 2022-02-25 | Carcass | Imjin River, Yeoncheon | EPI_ISL_18373247 |
| A/White-fronted goose/Korea/21WC260/2022 | 2022-02-28 | Carcass | Sampocheon Stream, Goseong | EPI_ISL_18373248 |
| A/White-fronted goose/Korea/21WC278/2022 | 2022-03-02 | Carcass | Cheorwon Plain, Cheorwon | EPI_ISL_18373249 |
| A/Bean goose/Korea/21WC279/2022 | 2022-03-04 | Carcass | Han River, Seoul | EPI_ISL_18373250 |
| A/White-fronted goose/Korea/21WC286/2022 | 2022-03-03 | Carcass | Songjiho Lake, Goseong | EPI_ISL_18373251 |
| A/Wild bird feces/Korea/21WF729-23/2022 | 2022-03-03 | Feces | Jeongeupcheon Stream, Jeongeup | EPI_ISL_18373252 |
| A/White-fronted goose/Korea/21WC300/2022 | 2022-03-07 | Carcass | Songjiho Lake, Goseong | EPI_ISL_18373253 |
| A/White-fronted goose/Korea/21WC305/2022 | 2022-03-04 | Carcass | Cheorwon Plain, Cheorwon | EPI_ISL_18373254 |
| A/White-fronted goose/Korea/21WC315/2022 | 2022-03-11 | Carcass | Jojongcheon Stream, Gapyeong | EPI_ISL_18373255 |
| A/White-fronted goose/Korea/21WC319/2022 | 2022-03-11 | Carcass | Songjiho Lake, Goseong | EPI_ISL_18373256 |
| A/White-fronted goose/Korea/21WC326/2022 | 2022-03-14 | Carcass | Songjiho Lake, Goseong | EPI_ISL_18373257 |
| A/White-fronted goose/Korea/21WC344/2022 | 2022-03-16 | Carcass | Songjiho Lake, Goseong | EPI_ISL_18373258 |
| A/Great egret/Korea/21WC347/2022 | 2022-03-14 | Carcass | Han River, Goyang | EPI_ISL_18373259 |
| A/White-fronted goose/Korea/21WC361/2022 | 2022-03-24 | Carcass | Songjiho Lake, Goseong | EPI_ISL_18373261 |

Supplementary Table 2. Divergence time of the most recent common ancestor of each genotype: N1.G1, N1.G1.1, N1.G2, N1.G2.1, and N1.G2.2

| Gene segment | N1.G1 | N1.G1.1 | N1.G2 | N1.G2.1 | N1.G2.2 |
| --- | --- | --- | --- | --- | --- |
|  | Mean tMRCA*  (95% HPD†) | Mean tMRCA  (95% HPD) | Mean tMRCA  (95% HPD) | Mean tMRCA  (95% HPD) | Mean tMRCA  (95% HPD) |
| PB2 | Nov 2019  (Apr 2019–  Apr 2020) | Jul 2021  (Feb 2021–Nov2021) | Nov 2020  (Jun 2020–  Mar 2021) | NA | NA |
| PB1 | Jan 2020  (Aug 2019–  Jun 2020) | NA | Jan 2021  (Sep 2020–  May 2021) | Dec 2020  (Jun 2020–  Jun 2021) | Jun 2021  (Sep 2021–  Jan 2022) |
| PA | Feb 2020  (Oct 2019–  Jul 2020) | NA | Nov 2020  (Mar 2019–  Nov 2020) | NA | NA |
| HA | Sep 2020  (Jul 2020–  Oct 2020) | NA | Jan 2021  (Nov 2020–  Apr 2021) | NA | NA |
| NP | Jan 2020  (Jul 2019–  Jul 2020) | NA | Aug 2020  (Mar 2020–  Dec 2020) | NA | NA |
| NA | Jan 2020  (Oct 2019–  Jun 2020) | NA | Feb 2020  (Oct 2019–  Sep 2020) | NA | NA |
| M | Apr 2020  (Nov 2019–  Aug 2020) | NA | Aug 2020  (Jul 2020–  Sep 2020) | NA | NA |
| NS | Mar 2020  (Sep 2019–  Aug 2020) | NA | Jul 2020  (Dec 2019–  Dec 2020) | NA | NA |

^*^Divergence time of the most recent common ancestor ^†^Highest posterior density. In case of genotype G2.2.1, tMRCA could not be calculated because of only one isolate in the cluster.

Supplementary Table 3. GISAID acknowledgment table. (Separate excel file)

**
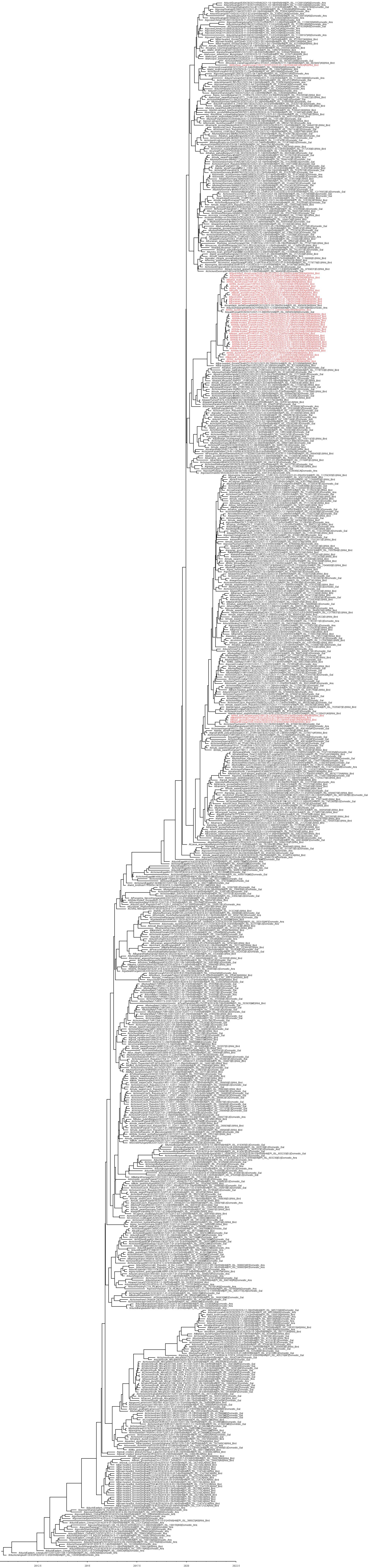
**

Supplementary Figure 1. The time scaled MCC tree of the HA gene segment of the 2.3.4.4b HPAI viruses with tip label. The viruses isolated in this study were indicated by red text.


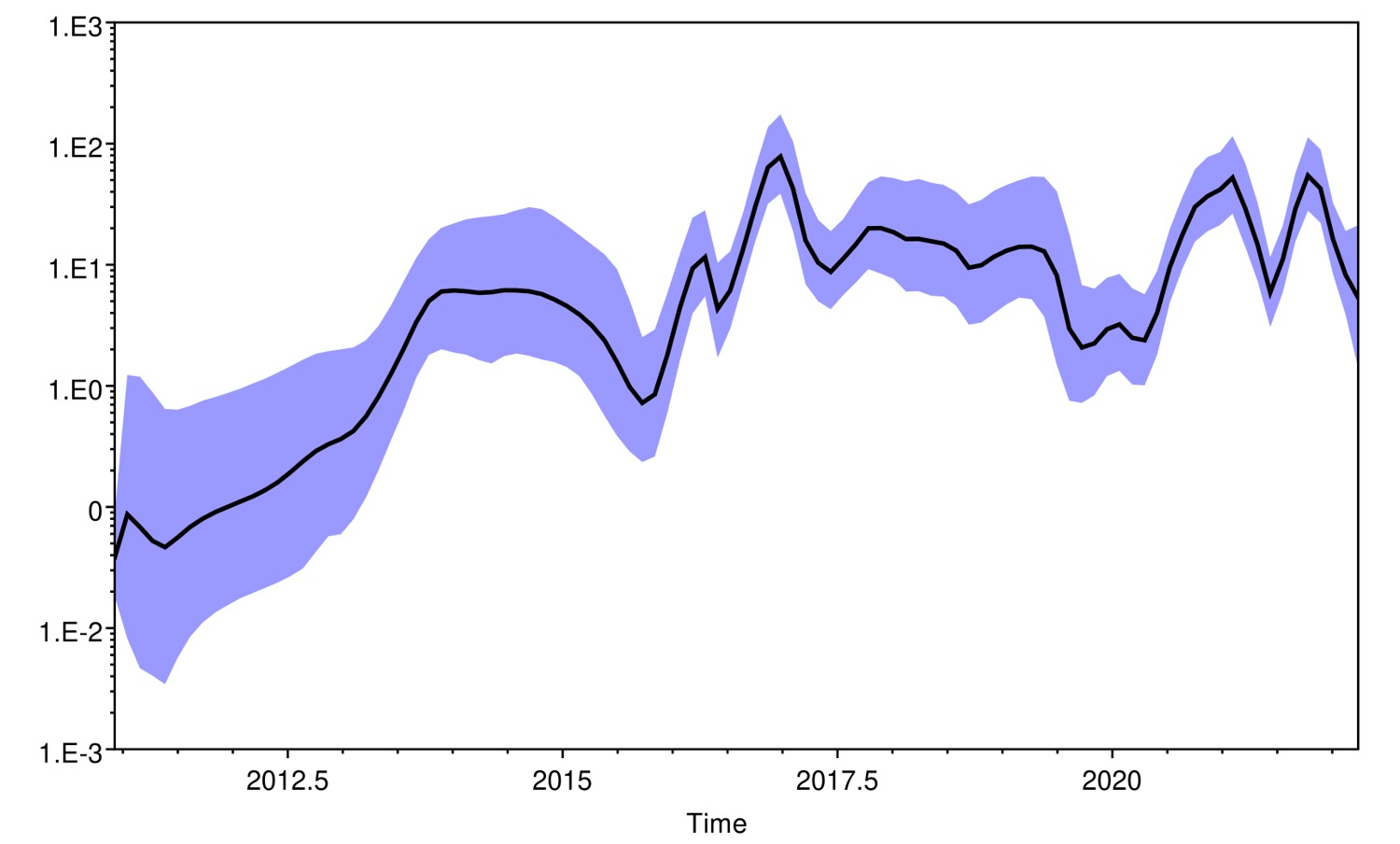


Supplementary Figure 2. GMRF Bayesian skyride plots indicating effective population size (relative genetic diversity) over time.


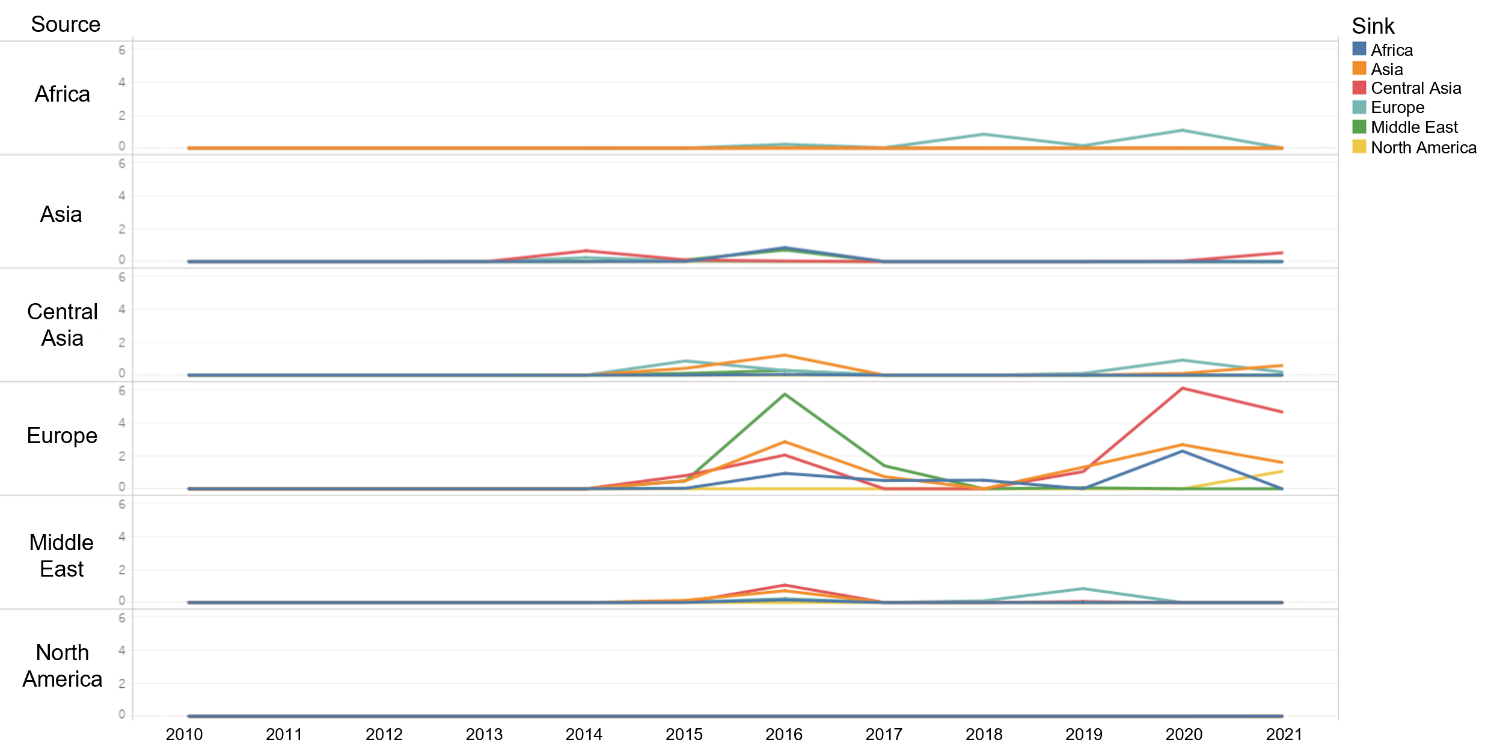


Supplementary Figure 3. A graph of the number of transition events between each geographical region by time zone.

**
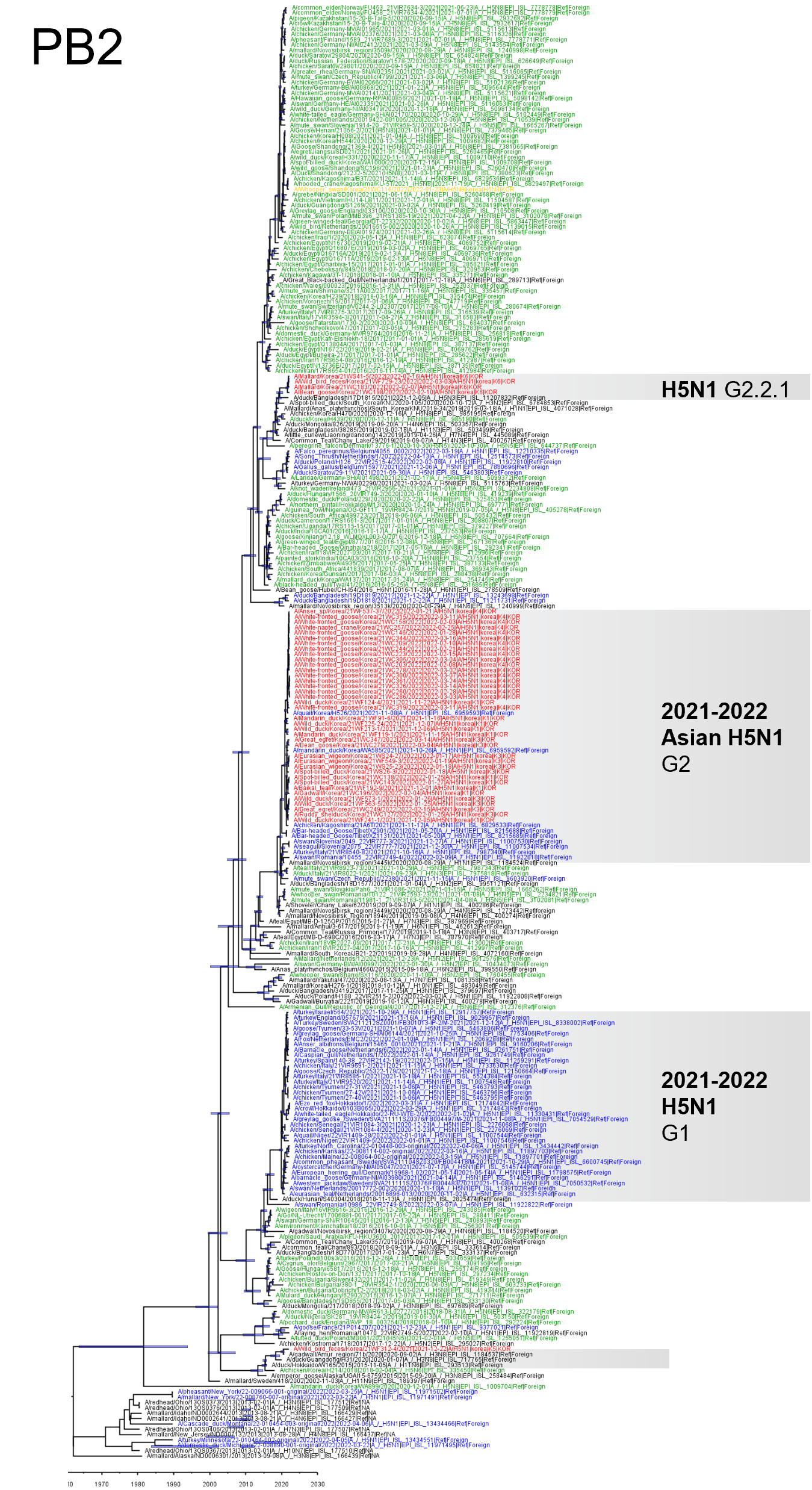
 (a)**

**
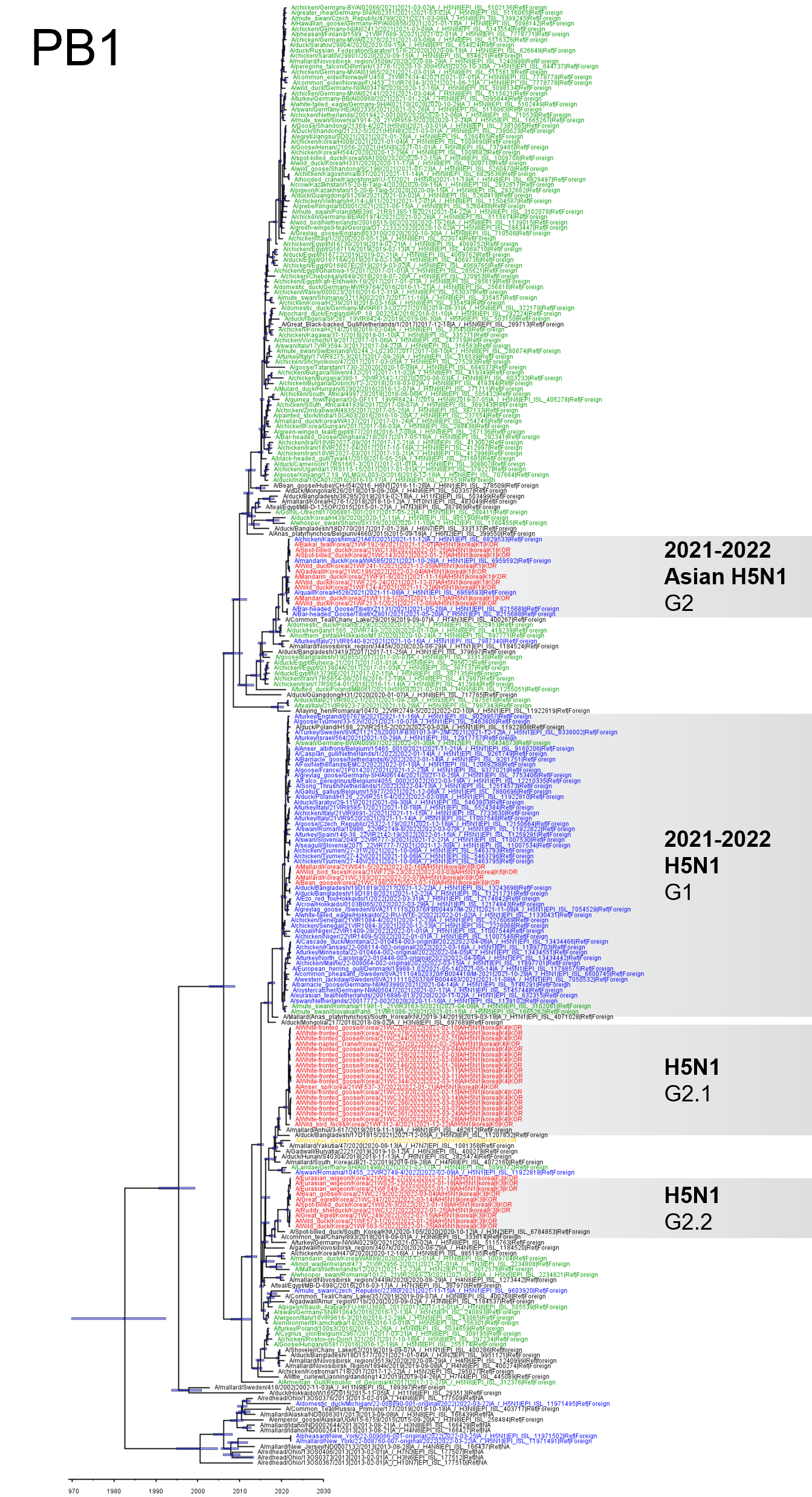
(b)**

**
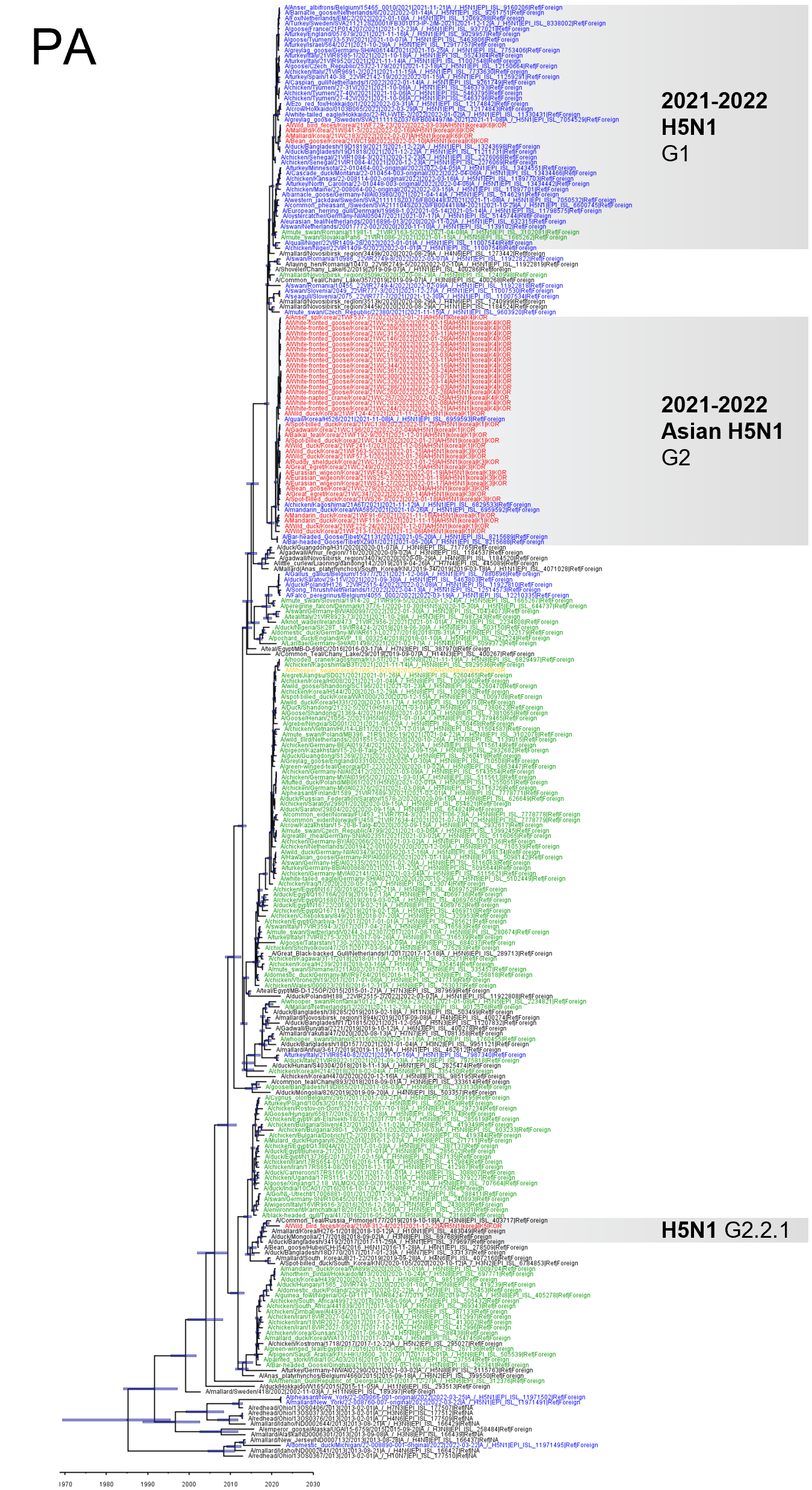
(c)**

**
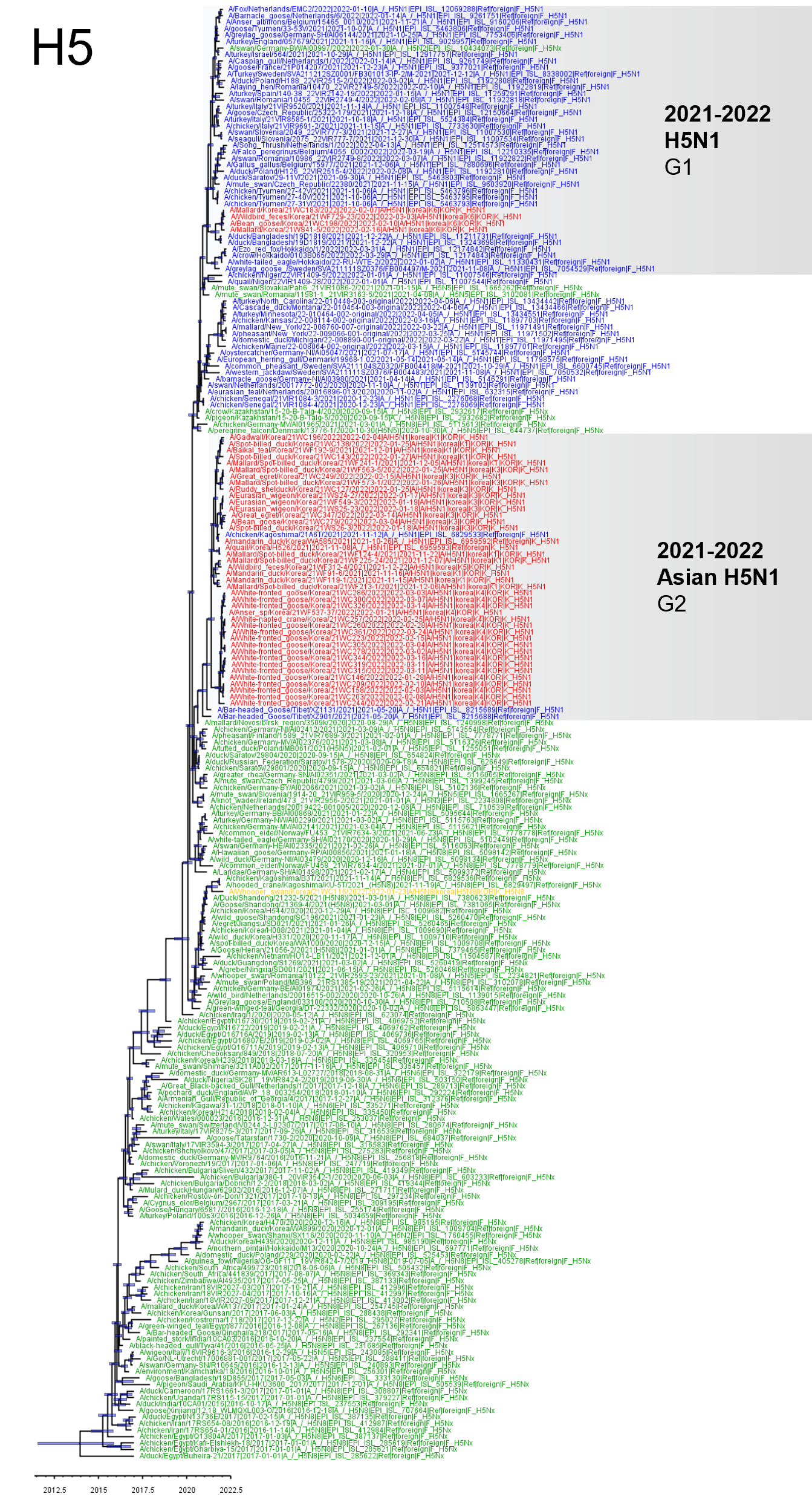
(d)**

**
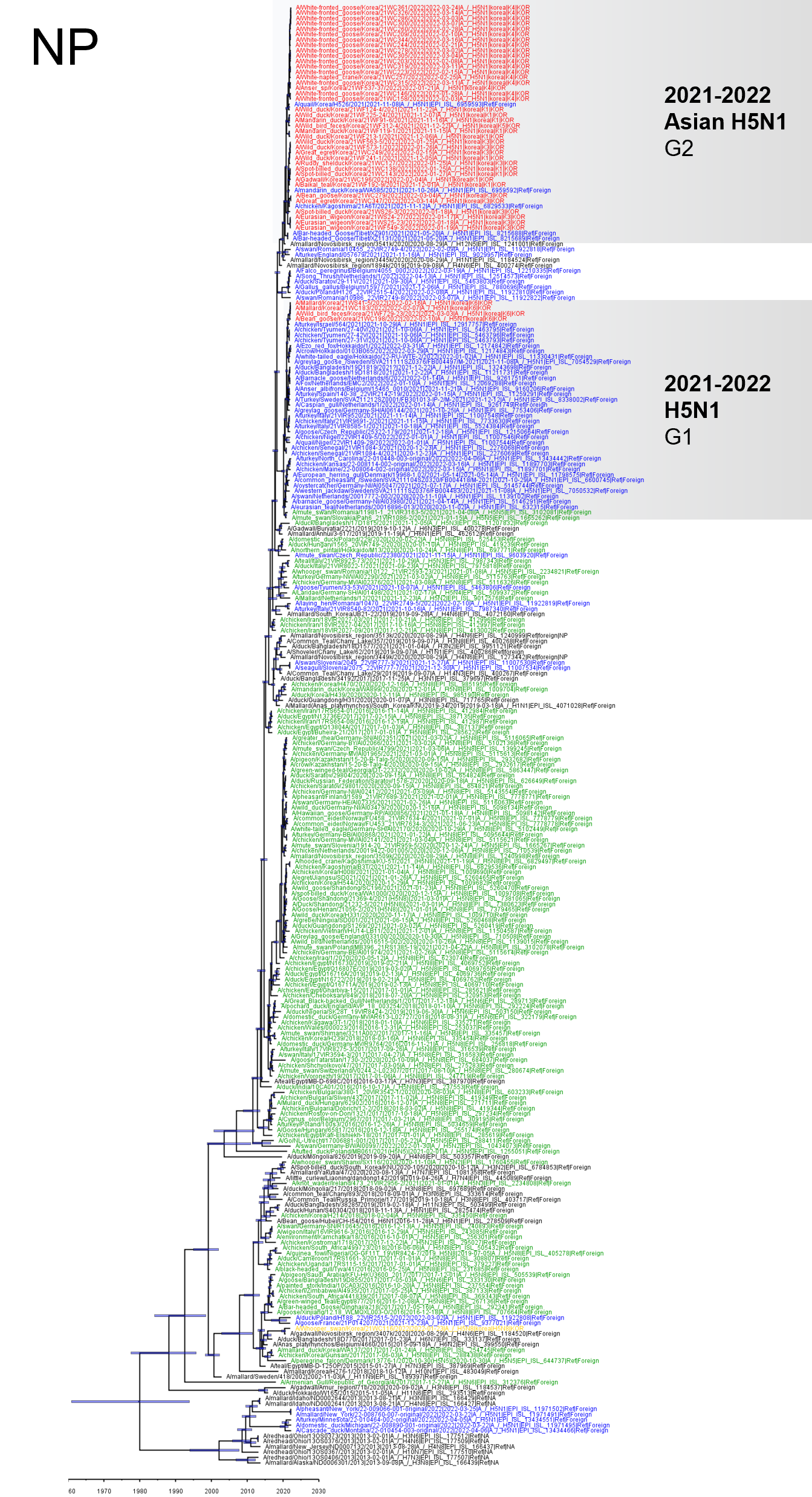
(e)**

**
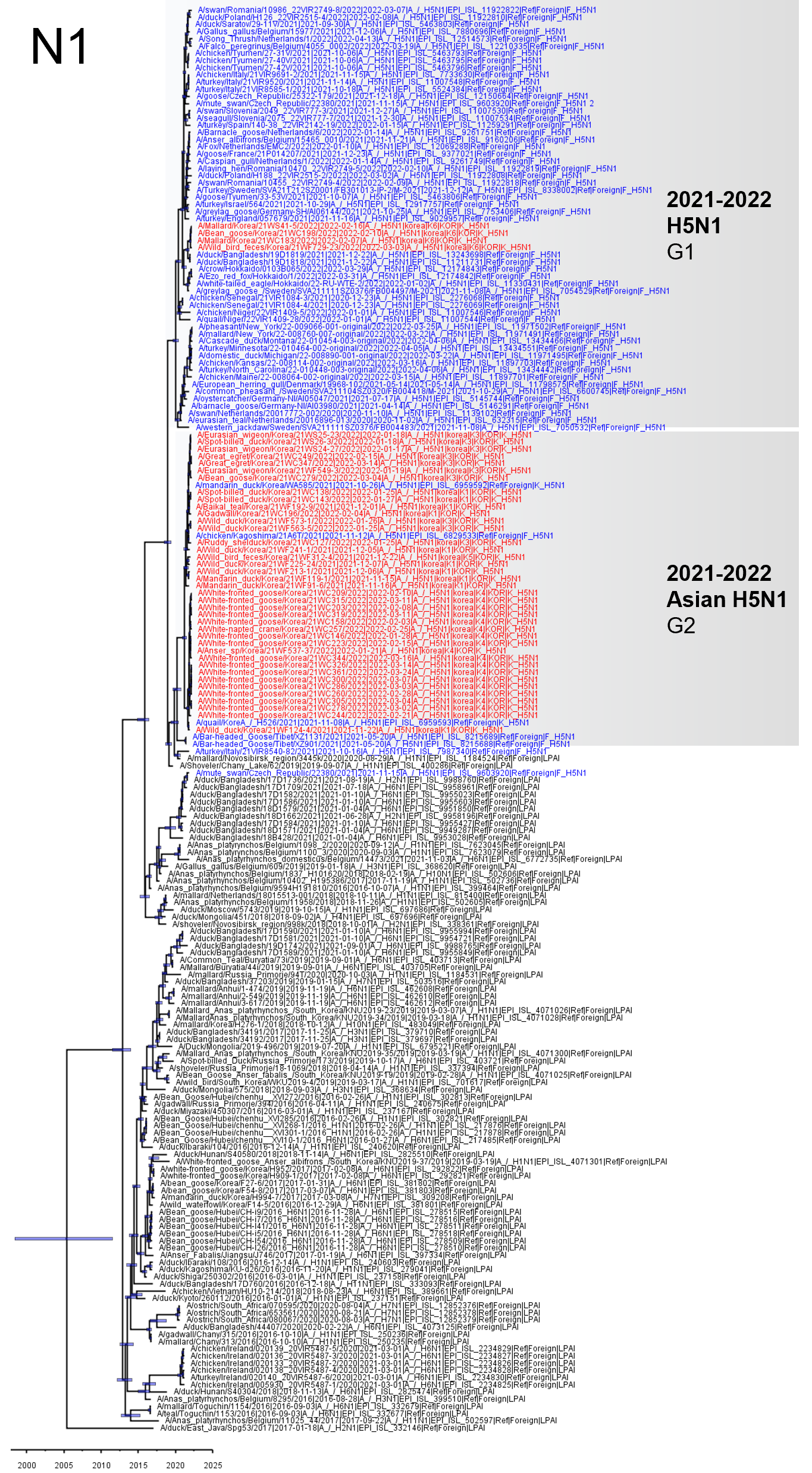
(f)**

**
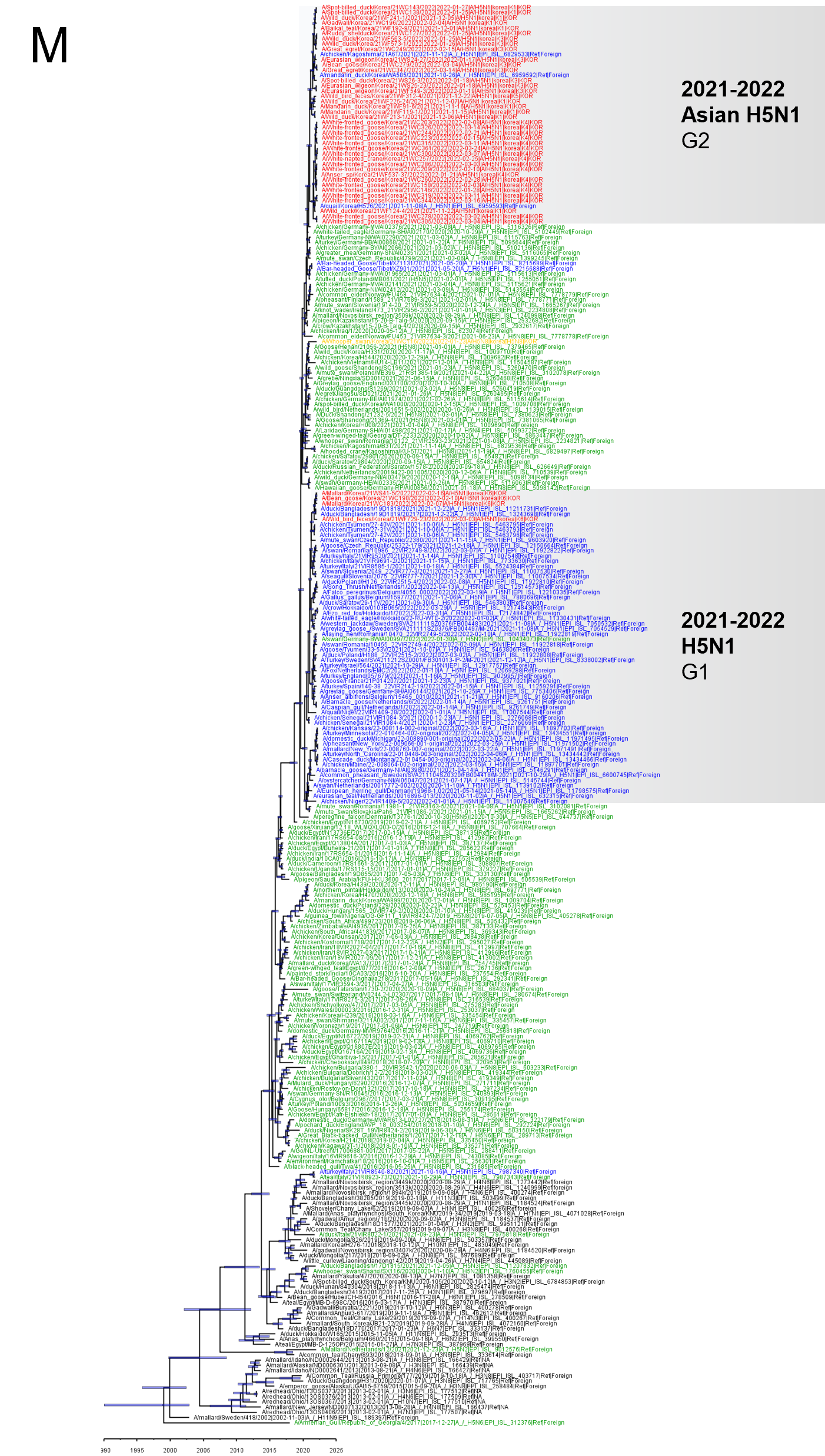
(g)**

**
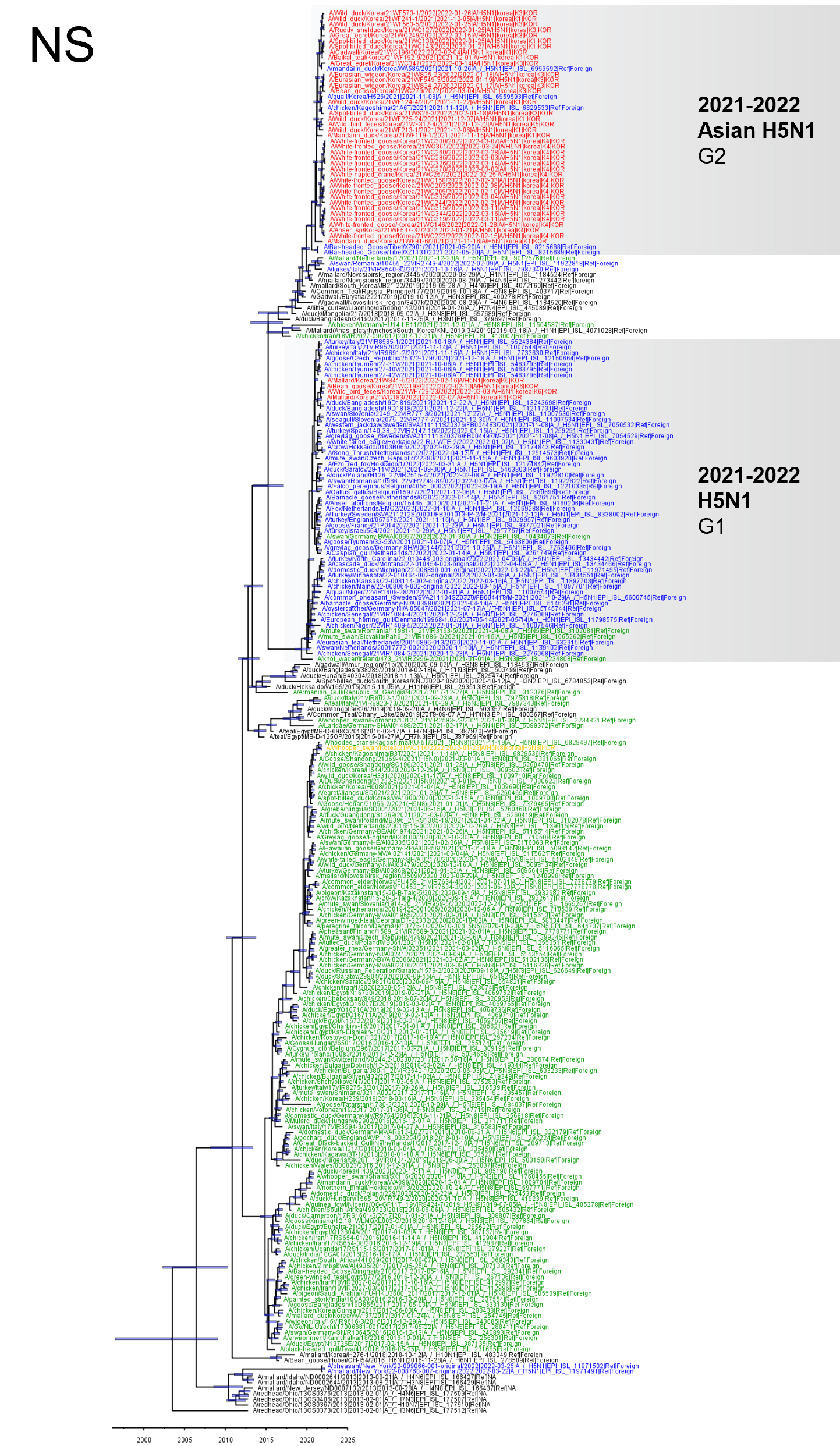
(h)**

Supplementary Figure 4. The time scaled MCC tree of each gene segment of viruses used to estimate tMRCA of each genotype. Korean H5N1 viruses indicated by red text. Korean H5N8 viruses indicated by yellow text. Foreign H5N1 viruses indicated by blue text. Foreign H5Nx viruses indicated by green text. Foreign LPAI viruses indicated by black text. We high-lighted each genotypic subgroup in gray box. (a) PB2, (b) PB1, (c) PA, (d) HA, (e) NP, (f) N1, (g) M, (h) NS.

Supplementary video 1. Spatiotemporal viral spread animation corresponding to Figure 3A. (Separate video file)
